# Optimizing HPV vaccine effectiveness: impact of vaccination age and dose schedule on immunogenicity and cervical cancer prevention

**DOI:** 10.3389/fpubh.2025.1544220

**Published:** 2025-05-07

**Authors:** Mehran Rostami Varnousfaderani, Zahedeh Khoshnazar, Hamidreza Zeratie, Parisa Hosseini Koukamari

**Affiliations:** ^1^Student Research Committee, Shahid Beheshti University of Medical Sciences, Tehran, Iran; ^2^Student Research Committee, Saveh University of Medical Sciences, Saveh, Iran; ^3^Department of Public Health, Saveh University of Medical Sciences, Saveh, Iran

**Keywords:** vaccine efficacy, dose optimization, public health, HPV, cervical cancer

## Abstract

Cervical cancer is the fourth most common cancer among women globally, claiming over 443,000 lives annually, with 98% of these deaths occurring in developing countries. Vaccination against human papillomavirus (HPV) is a preventive strategy. This review investigates the role of age at vaccination and the number of doses in determining vaccine effectiveness. Articles from 2013 to 2023 were retrieved from PubMed, Scopus, SID, and Google Scholar using keywords related to HPV, vaccine, age, and dose. The findings suggest that the highest vaccine effectiveness is observed in younger age groups (ages 9–14: 74–93%) and decreases with age. Studies indicate that while three doses provide optimal protection, a single dose may also confer significant benefits in younger populations. These findings underscore the importance of timely vaccination and adherence to dosing schedules for maximizing vaccine impact.

## Introduction

HPV is part of the most prominent family of sexually transmitted viruses (STDs) that infect a very large number of people annually (1). From a virological perspective, the human papillomavirus is a small double-stranded DNA virus and belongs to the group of nonenveloped icosahedral viruses (2). More than 200 genotypes of this virus have been identified and detected (3). Some of these genotypes, such as genotypes 6, 11, 40, 42, 43, 44, 54, 61, and 72, usually cause benign lesions, such as warts in different parts of the body, such as the head, neck, and urogenital tract, which are called low-risk types (4, 5). On the other hand, types 16, 18, 31, 33, 35, 39, 45, 51, 52, 56, 58, 59, 66, and 69 are among the common high-risk types that cause cancer (6). Among the high-risk HPV types, HPV-16 and HPV-18 are known as the etiological agents of cervical cancer, accounting for approximately 70% of the burden of this cancer (7). The virus infects both males and females, but in most cases, it is asymptomatic and does not cause any particular disease; usually, after 1–2 years, it is self-cured and eliminated from the body (8). Initially, the infection starts with damage to the mucosal and skin tissues of the desired area and causes the formation of a wound or wart at the site. This virus usually affects the epithelial tissue of the genital area, especially in women (9). If the host immune system fails to clear the HPV infection, high-risk types of, HPV can activate oncogenes in the body (10).

Cervical cancer is the third most common malignancy of the female genital tract, and according to the World Health Organization, cervical cancer is the fourth most common cancer among women (11). More than 90% of cervical cancers and precancerous lesions are associated with HPV (12). The organization also stated that by 2030, cervical cancer will affect the lives of more than 443,000 women worldwide, and 98% of these deaths will occur in developing countries (13). Cervical cancer is the second leading cause of cancer death among women in developing countries (14). The International Agency for Research on Cancer (IARC) reported that in 2020, 604,000 women worldwide were diagnosed with cervical cancer, and approximately 342,000 women died from the disease (15).

The most important way to prevent cervical cancer is to prevent HPV infection. Three types of vaccines are available to prevent this viral infection: the bivalent type is effective against types 16 and 18 of the virus. The quadrivalent type covers types 11 and 6 in addition to these two types. The 9-valent HPV vaccine (Gardasil 9), which is recommended for individuals aged 9–45 in the United States, protects against HPV types 31, 33, 45, 52, and 58 in addition to the four main types covered by earlier versions. This vaccine is approved for use in both females and males to prevent HPV-related diseases, including cervical, vulvar., vaginal, anal, and oropharyngeal cancers, as well as genital warts (16, 17). The present study aimed to investigate the effectiveness of the human papillomavirus vaccine based on the age at which it was vaccinated and the appropriate number of doses.

In this review, our goal was to provide an overview of the effectiveness of the appropriate dose of HPV vaccination and the appropriate age for receiving the vaccine, which was conducted via a narrative review method. The search process was carried out from 2013 to 2023 in PubMed, Scopus, SID, and Google Scholar search engines by entering keywords such as human papillomavirus or HPV, age, dose, papilloma vaccine (HPV vaccine), and cervical cancer. In PubMed, the subject search was performed via MeSH. In the mentioned databases, a comprehensive and accurate review of published articles on vaccination for the prevention of cervical cancer and the role of age and vaccine dose in the effectiveness of this vaccine was conducted. In this context, articles that met the inclusion criteria were selected. The inclusion criteria for this study included the following: 1. The search words and keywords were included in the title and keywords of the article. 2. Full access to the text of the articles, including tables and figures, was available. 3. The focus of the article was on the number of doses received and the age of vaccination. Incomplete articles that were off-topic and did not have full access to the text were excluded from the study.

In this review, keywords were searched in the PubMed, Scopus, SID, and Google Scholar search engines, with a time limit of 2013–2023. The search keywords were as follows: human papillomavirus, age, dose, papilloma vaccine (HPV vaccine), and cervical cancer. The subject search was performed through *MeSH*. The inclusion criteria were articles focused on age and dose-specific vaccine effectiveness, full-text availability, and published in English.

## Assumptions underlying vaccine efficiency analysis

The assumptions used in this review about the effectiveness of HPV vaccines are based on a number of key factors that could influence the extent to which vaccination is effective. First, it is assumed that vaccination at an earlier age, particularly before exposure to the virus (i.e., before sexual activity)—will elicit a stronger immune response, as this group is less likely to have been previously infected with HPV. This assumption is supported by the results of studies showing that 9- to 14-year-olds have higher antibody titers and greater levels of protection against HPV-related diseases than older adolescents (15–18-year-olds).

The second assumption is that completing the full vaccination schedule (two or three doses) provides optimal protection against HPV infection and related cancers. Although some studies suggest that even one dose may be sufficient for younger people, this is based on the assumption that more doses provide stronger and more lasting immunity, especially in older people or those who may have been previously exposed to the virus.

Another assumption is that vaccines such as Gardasil and Cervarix are able to provide cross-protection against different HPV types and that their effectiveness is, overall, comparable. However, some evidence suggests that the bivalent vaccine Cervarix may provide greater cross-protection against HPV types other than 16 and 18.

On the other hand, this review is based on the assumption that vaccination at an early age is not only more clinically effective but also more economically viable, as it leads to reduced future costs of treating HPV-related diseases, including cervical cancer. It is also hypothesized that single-dose vaccination strategies could simplify implementation, reduce costs, and increase vaccination coverage in resource-limited settings.

However, the review still emphasizes that completing the full vaccination regimen (two or three doses) is recommended for optimal protection, especially in older individuals, as this group may require more doses to achieve the same level of protection as younger individuals.

Finally, 17,790 records were found. A total of 992 articles were reviewed, and after irrelevant, duplicate, and noncompliant articles meeting the inclusion criteria were excluded, 28 articles were ultimately selected. Table 1 presents an overview of studies on HPV vaccine efficacy, with a particular focus on demographic factors and the development status of the countries studied. These studies show distinct differences between developed and developing countries in terms of target populations, vaccination schedules, and vaccine effects in different age groups. In developed countries, the main focus has been on the efficacy of multiple doses and age-related responses, and studies have mostly focused on adolescent girls and young women. In contrast, studies in developing countries have emphasized the potential for more cost-effective options, such as single-dose regimens, to increase vaccination coverage and reduce cervical cancer incidence. These studies suggest that demographic factors, such as age at vaccination and access to resources, play a key role in determining the best vaccination strategies. The results indicate that the effectiveness of the vaccine is greater among younger age groups and that the design of vaccination programs should be tailored to the specific characteristics and challenges of each country in terms of demographic structure and economic conditions in order to have the greatest impact on public health.

**Table 1:** Purpose, outcome, and demographic distribution considerations in HPV vaccine effectiveness studies based on the number of vaccine doses and development status.

Number	Author Name	Country	Development Status	Year	Aim	Demographics factor	Result
1.	M. K. Ellingson	USA	Developed	2023	Efficacy of human papillomavirus vaccine according to age at the time of vaccination	Female, age-specific analysis (e.g., <15 vs. ≥ 15), multiple studies included	HPV vaccine at younger ages is effective against HPV-related disease outcomes. Human papillomavirus (HPV) vaccines work by preventing infections before natural exposure.
2.	Waheed, D. E	Global (Meeting in Belgium)	Developed	2023	Update on studies of single-dose HPV vaccination and humoral immune responses after HPV vaccination.	Females aged 9–20 years (target population for one-dose schedule)	There is no significant relationship between the number of vaccine doses and their effectiveness.
3.	Villa L. L	Brazil	Developing	2023	Investigating the time to optimize programs and recommendations about reducing vaccine doses from three doses to two doses in low and middle-income countries.	Girls and adolescents (9–14), some adults with immunodeficiency	Significant effect of single-dose vaccination on HPV reduction with lower cost and simpler implementation
4.	I. Man	India	Developing	2023	Determining the health and economic effects of introducing single-dose or two-dose human papillomavirus vaccination in India	Girls aged 10; national and state-level modeling based on Indian population data	Single-dose vaccination is more effective than no vaccination, but there is no difference between vaccination with one dose and vaccination with two doses in terms of effectiveness.
5.	K. Prem,	Global (188 countries)	Developed & Developing countries	2023	Determining the long-term health benefits and cost-effectiveness of single-dose versus two-dose HPV vaccination	Girls aged 10; global model with catch-up to age 14 at 80% coverage	One-dose vaccination has similar health benefits to a two-dose schedule while simplifying vaccine delivery, reducing costs, and reducing vaccine supply constraints.
6.	T. Gheit	India	Developing country	2023	To determine the effect of HPV vaccination on HPV-related oral infections	818 vaccinated and 179 unvaccinated sexually active women	A single dose of the vaccine is less effective than two or three doses in preventing oral HPV infection.
7.	É. Bénard	India, Nigeria, Uganda, Vietnam	Low & Middle-income countries	2023	Determining the potential efficacy of single-dose HPV vaccination at the population level in low-and middle-income countries	Girls aged 9–14; modeled using real datasets	Vaccination with one dose can prevent many cervical cancers that can be prevented by vaccination with two doses while being more effective.
8.	L. E. Markowitz	Multiple	Mixed-income countries	2022	Determination of HPV vaccine efficacy based on number of doses in up-to-date observational studies	Mostly adolescent girls; age at vaccination a key factor	Most studies obtained the highest efficacy estimate with three doses and showed greater efficacy at a younger age.
9.	F. Nicoli	Italy	Developed	2022	Effects of age of vaccination on humoral response to human papillomavirus vaccine	315 females stratified by age: adolescents vs. adults	Adolescents respond better to the 4vHPV vaccine than adults.
10.	P. Basu	India	Developing	2021	Determination of vaccine efficacy against persistent human papillomavirus (HPV) 16/18 infection at 10 years after one, two, and three doses of quadrivalent HPV vaccine in girls in India	Unmarried girls aged 10–18, followed into adulthood	One dose of HPV vaccine provides similar protection against persistent infection with HPV 16,18 genotypes responsible for approximately 70% of cervical cancers as two or three doses.
11.	C. Acuti Martellucci	Italy	Developed	2021	Determining the effectiveness of the human papillomavirus vaccine in cervical cancer screening programs	Women born 1986–1993, participated in screening 2011–2018 (mean age 27.5)	Women receiving at least one vaccine dose were significantly less likely to have abnormal cytology, and there was no difference between the number of vaccine doses and the prevention of abnormal cytology.
12.	M. K. Abel	USA	Developed	2021	Determining the prevalence of oral human papillomavirus infection by number of vaccine doses among US adults	Adults from NHANES 2009–2016; smokers, early oral sex initiators, multiple partners	Determining the prevalence of oral human papillomavirus infection by number of vaccine doses among US adults
13.	P. Wnukowski-Mtonga	Australia	Developed	2020	Determining the scientific evidence supporting recommendations for the use of the nine-valent HPV vaccine in the 2-dose vaccine program in Australia.	Adolescents aged 9–14 years; comparisons with women aged 16–26	For all three HPV vaccines, immunogenicity data (comparison of seroconversion and antibody titers) show that the 2-dose schedule in adolescents aged 9 to 14 years is noninferior to the 3-dose schedule in young adults aged 16 years and older.
14.	L. E. Markowitz	United States	Developed	2020	Determining the effectiveness of the HPV vaccine against the prevalence of HPV with the number of doses and the age of receiving the vaccine received.	Women aged 20–29 years; focus on age at first dose (≤18 years)	The prevalence of vHPV type 4 among unvaccinated women was 7.4% compared with 1.7, 1.0, and 1.0% among those receiving 1, 2, and 3 doses and also among women who received their first dose at age ≤18. HPV vaccine was high regardless of the number of doses.
15.	N. Bhatla	India	Developing	2020	Providing recommendations for HPV vaccination in India.	Girls <15 years, women 9–45 years, immunocompromised individuals, survivors of sexual assault, older women	HPV vaccination is recommended for all girls under 15 years of age as the best target group. Two doses 6 months apart, extendable up to 18 months, is the best option, but the results of single-dose vaccination are promising.
16.	R. Murillo	Low-and middle-income countries	Developing	2019	Determination of HPV vaccine efficacy reported in clinical trials and population-based studies	HPV-negative young women (<25 years) and adult women for catch-up vaccination	HPV vaccines are nearly 100% effective when used in a three-dose schedule in young women (less than 25 years of age) to protect against persistent infection and precancerous lesions associated with the type of HPV vaccine.
17.	J. M. Brotherton	Australia	Developed	2019	Determining whether one dose of human papillomavirus vaccine is as effective as three doses?	Women aged 15 or younger at vaccination, up to 7 years post-vaccination	One dose had comparable efficacy to two or three doses in preventing high-grade disease in a high-coverage setting. So that, there was no difference between the 1st, 2nd and 3rd doses in terms of the adjusted risk ratio compared to the nonvaccinated group.
18.	P. K. Braverman	United States	Developed	2019	Determining the effectiveness of the HPV vaccine in adolescents	Adolescents, particularly those receiving their first dose before age 15	Because higher titers are seen at younger ages, two doses are needed instead of three if the first dose is given before age 15.
19.	H. Bergman	Global	Mixed	2019	Comparison of different types of human papillomavirus (HPV) vaccine and dosing schedule for the prevention of HPV-related disease in women and men	Females and males aged 9–26, including those with HIV	Two versus three doses of HPV vaccine in 9- to 15-year-old females Antibody responses after two-dose and three-dose HPV vaccine schedules were similar after 5 years of follow-up.
20.	R. Sankaranarayanan	India	Developing	2018	Determining whether a single dose of human papillomavirus (HPV) vaccine can prevent cervical cancer.	Unmarried girls aged 10–18 years	Our results show that a single dose of quadrivalent HPV vaccine is immunogenic and provides sustained protection against HPV 16,18 infections similar to three-dose vaccine schedules.
21.	L. Lin	Colombia, Mexico, Panama	Developing	2018	Determination of immunogenicity of HPV-16/18 AS04 adjuvanted vaccine in 4-6-year-old girls	Healthy girls aged 4–6 years	Two-dose vaccination with AS04-HPV-16/18 produces sufficient antibody response in 4-6-year-old girls.
22.	T. Võrno	Estonia	Developed	2017	Determining the cost-effectiveness of HPV vaccination in the context of high cervical cancer incidence and low screening coverage	12-year-old girls	Vaccination of 12-year-old girls alongside current cervical cancer screening could be considered a cost-effective intervention in Estonia.
23.	A. M. Hofstetter	United States	Developed	2016	Determination of human papillomavirus vaccination and cervical cytology results among low-income urban minority women	Female patients aged 11–20 years, low-income, primarily Spanish-speaking, publicly insured.	The risk of an abnormal cervical cytology result was lower among vaccinated women than unvaccinated women, especially if the 3-dose series was completed or if the vaccine was administered from 11 to 14 years of age.
24.	S. Aljunid	Malaysia	Developing	2016	Determining the cost-effectiveness of an HPV vaccination regimen: comparing two versus three doses of vaccination in adolescent girls in Malaysia	13-year-old school girls in Malaysia	A 2-dose HPV vaccination plan may enable Malaysian women to be protected at a lower cost than a 3-dose plan while preventing a similar number of cervical cancer cases and deaths.
25.	Z. Q. Toh	Australia	Developed	2015	Reduced-dose human papillomavirus vaccination: an update of the latest current status	Adolescents (9–15 years old) and women (>15 years old)	There was no difference in antibody response between adolescents (9–15 years) who received two doses (6 months apart) and females (over 15 years) who received the standard three-dose schedule.
26.	L. Mariani	Australia, New Zealand, United States, Denmark, Germany, Sweden	Developed	2015	Determining the primary direct and indirect effect of quadrivalent HPV (4HPV) vaccine on genital warts	Women aged <21 years, school-based and non-school-based vaccination programs, vaccine uptake rates (VUR) of 70% for 3 doses	Vaccine protection is more likely at a younger age.
27.	E. Herweijer	Sweden	Developed	2014	To determine the association between quadrivalent HPV vaccination and the first occurrence of condyloma about vaccine dose in a population-based setting.	Females aged 10–24 years, stratified by age at first vaccination	Although the maximum reduction in the risk of condyloma was observed after receiving 3 doses of the quadrivalent HPV vaccine, receiving 2 doses of the vaccine was also associated with a significant reduction in the risk of condyloma.
28.	S. R. Dobson	Canada	Developed	2013	To determine whether mean HPV-16 and HPV-18 antibody levels are lower in girls receiving 2 doses than in women receiving 3 doses.	Girls (9–13 years) and young women (16–26 years)	Among girls who received 2 doses of HPV vaccine 6 months apart, responses to HPV-16 and HPV-18 were lower 1 month after the last dose than among young women who received 3 doses of vaccine within 6 months.

### Effectiveness of the vaccine based on the number of doses received

Several studies have shown a relationship between the number of vaccine doses and their effectiveness and immunogenicity, and the responses from a single dose of HPV vaccination result in lower antibody titers in the serum (18). A single vaccine dose may be less effective than two or three doses in preventing oral HPV infection (19), and the maximum effectiveness of the vaccine is achieved by receiving three doses (20).

On the other hand, some studies have shown no difference between the number of vaccine doses and the vaccine effectiveness (21–24). A single dose of the HPV vaccine provides similar protection against persistent infection from HPV genotypes 16 and 18, which are responsible for nearly 70% of cervical cancers, as two or three doses (25). Single-dose HPV vaccination can significantly reduce the incidence of precancerous and cervical cancers attributed to HPV, with reduced costs for vaccine delivery and simpler implementation, allowing more countries to introduce HPV vaccination or increase compliance in the target population. Although it does not differ in its ability to induce immunity at two doses, it is more effective (26, 27). Global introduction of one dose of HPV at 10 years of age has been presented as a cost-saving policy, particularly in the low-and middle-income context where vaccine cost and availability are a huge consideration (28). Evidence is such that the immune response provided by one dose is found to be extremely intense and long-lasting, yet any marginal value created by increasing doses from one may not be a reason for an added logistical expense and burden incurred (29). Single-dose vaccination with Gardasil 9 (9-valent) has similar health benefits to two-dose vaccination while simplifying vaccine delivery, reducing costs, and alleviating vaccine supply constraints. The second dose may be cost-effective if there is a shorter duration of protection from one dose, a cheaper vaccine, vaccination delivery strategies, and a high burden of cervical cancer (30).

Besides the dosing number, the formulation of the HPV vaccine used may contribute to the modulation of the immune response and vaccine efficacy. Other studies have pointed out that the bivalent vaccine (Cervarix) is more likely to induce a better and more durable antibody response against HPV 16 and HPV 18 than are the quadrivalent and 9-valent vaccines, particularly after single-dose regimens, due to reasons presumably owing to its adjuvant system (31, 32). However, the 9-valent vaccine (Gardasil-9) has broader coverage by targeting an additional five oncogenic HPV types, potentially for use in the presence of more disseminated circulating genotypes (33). Although few direct comparisons have occurred, evidence on hand suggests vaccine type choice will moderately influence immune response in regimens using one dose, though more research must be done in order to render absolute judgments.

In general, women who receive one dose do not differ in terms of abnormal cervical cytology from women who receive two or three doses (34), and individuals who receive one dose of the HPV vaccine may have a similar prevalence of oral HPV6, 11, 16, and 18 infections as those who receive additional doses (35).

Additionally, other studies have shown that a two-dose vaccination program does not have lower immunogenicity than a three-dose vaccination does (36–38), and two doses with a 6-month interval, which can be increased to 18 months, are the best option (39). Vaccination with two doses induces a sufficient antibody response (40) and prevents a similar number of cervical cancers and deaths at a lower cost than three doses (41). However, some studies have shown that among girls who received two doses of the HPV vaccine 6 months apart, the response to HPV-16 and HPV-18 one month after the last dose was lower than that in young women who received three doses of the vaccine within 6 months (42).

### Effectiveness of the vaccine based on age at vaccination

Most studies have shown greater effectiveness when individuals are vaccinated at a younger age (20–28, 30, 34–43). The younger the subjects are at the time of vaccination, the less likely they are to have been exposed to HPV; therefore, they are more likely to be protected by preventive vaccines such as the HPV4 vaccine (39). In other words, higher titers are produced at younger ages, and if the first dose is given before the age of 15, one dose of the vaccine is needed instead of three doses (44). Based on a systematic review including 21 individual studies, the reported vaccine effectiveness ranged from approximately 74–93% among adolescents aged 9–14 years, and from 12 to 90% among those aged 15–18 years. These wide ranges reflect substantial variability in study populations, methodologies, and outcome definitions. These results suggest that the HPV vaccine is more effective against HPV-related disease outcomes when administered at younger ages and emphasizes the importance of timely vaccination (45).

On the other hand, the percentage of naive B and CD4 + T cells was significantly greater in adolescents, and the latter correlated directly with IgG titers against 3 of the 4 HPV types. HPV-specific IgGs, but not memory B cells, are induced and maintained at relatively high levels in individuals vaccinated during adolescence (46). Among women who received their first dose under the age of 18, the estimated effectiveness of the HPV vaccine was high, regardless of the number of doses (21). The target group under 15 and 11–14 years of age is the best target group for receiving the vaccine (39, 47). In general, vaccination with 3 doses in women under 25 years of age has an effectiveness close to 100% in protecting against persistent infection (48), and vaccinating 12-year-old girls, along with screening, can be considered an effective intervention (49). Some studies have also shown that for all three HPV vaccines, the immunogenicity data (comparison of seroconversion and antibody titers) indicate that they are not lower in adolescents aged 9–14 years than in young adults aged 16 years and older (36).

The present study investigated the effectiveness of the human papillomavirus vaccine on the basis of the number of doses and the appropriate age at vaccination. The presence of papillomaviruses can lead to cervical cancer (12). Cervical cancer can be controlled through screening, vaccination, and safe sexual relationships. To date, on the basis of the existing serotypes, three types of vaccines have been developed against this virus: Cervarix (bivalent), Gardasil (quadrivalent), and Gardasil-9 (9-valent) are the three vaccines developed against HPV (16, 17). Cervarix is a vaccine capable of protecting against types 16 and 18, which cause 91% of cervical cancers, 91% of anal cancers, and 61% of vaginal cancers (50). The Gardasil vaccine protects against four HPV serotypes, namely, 6, 11, 16, and 18. The Gardasil-9 vaccine contains particles of serotypes 6, 11, 16, 18, 31, 33, 45, 52, and 58 and induces immunity against these serotypes (51). Despite being bivalent, the efficacy and coverage of the Cervarix vaccine are significantly greater than those of the quadrivalent Gardasil vaccine, demonstrating the high potential of the Cervarix vaccine in providing cross-protection (50).

The reviewed studies consistently show that HPV vaccination is more effective when it is administered at younger ages, typically before exposure to HPV and the initiation of sexual activity (43, 45). Vaccination during preadolescence and early adolescence (e.g., ages 9–14) resulted in the highest rates of seroconversion, antibody titers, and protection against HPV infection and related diseases (39). Vaccination in older adolescents and young adults (e.g., ages 15 to 26) is still beneficial but has slightly lower effectiveness than in younger individuals (48).

These data indicate that a complete series of 2 or 3 doses of HPV vaccines is required to achieve optimal protection (24, 38). Receiving fewer than recommended number of doses was associated with decreased antibody levels and lower effectiveness against HPV infection and related diseases (38). However, some studies have shown that even a single dose of the HPV vaccine provides meaningful protection, particularly in younger age groups (22, 25). A study by Aljunid et al. (41) revealed that the two-and three-dose regimens of the HPV vaccine had similar effects on controlling cervical cancer, and using the two-dose regimen resulted in lower costs and was more cost-effective (41).

This review highlights that younger age at vaccination and adherence to dosing schedules are critical for maximizing HPV vaccine effectiveness. These findings align with global recommendations advocating vaccination before exposure to HPV, typically before the onset of sexual activity.

Despite the strengths of this study, its limitations include the following: Heterogeneity: Variability in study designs, populations, and endpoints limits direct comparisons of findings. Data gaps: Inadequate data on the long-term effectiveness and immunity in single-dose recipients call for additional studies. A review of published research from 2013 to 2023 indicates that protection against single-dose immune response and single-dose protection have persisted for up to 10 years. However, additional follow-up studies are necessary to ascertain if such protection can persist over a decade.

## Conclusion

Since cervical cancer is a common cancer, preventing it is considered important. Accordingly, preventing HPV infection forms the basis and foundation of recent research. Cumulative evidence emphasizes the vital role of the HPV vaccine in preventing HPV infections and related diseases, especially cervical cancer. With the help of vaccination at an appropriate age, many precancerous and cancerous lesions of the female genital area can be prevented. HPV vaccination is a pivotal strategy for reducing the incidence of cervical cancer, particularly in resource-limited settings. Early administration and appropriate dosing maximize immunogenicity and cost-effectiveness. Public health campaigns should emphasize timely vaccination and adherence to recommended schedules. Even in developing or underdeveloped countries, appropriate vaccination doses against this virus can be administered cost-effectively. On the other hand, educating and raising awareness among individuals in society will play a clear and crucial role in the successful and timely (appropriate age) administration of vaccination. By adhering to the recommended number of doses on the basis of age groups and initiating vaccination at younger ages, healthcare providers can maximize the effectiveness of the vaccine in providing long-term protection against HPV-related health risks.
